# An Updated Mendelian Randomization Analysis of the Association Between Serum Calcium Levels and the Risk of Alzheimer’s Disease

**DOI:** 10.3389/fgene.2021.731391

**Published:** 2021-09-08

**Authors:** Yuchen Shi, Ruifei Liu, Ying Guo, Qiwei Li, Haichun Zhou, Shaolei Yu, Hua Liang, Zeguang Li

**Affiliations:** ^1^Heilongjiang University of Chinese Medicine, Harbin, China; ^2^Second Affiliated Hospital, Heilongjiang University of Chinese Medicine, Harbin, China; ^3^First Affiliated Hospital, Heilongjiang University of Chinese Medicine, Harbin, China

**Keywords:** Alzheimer’s disease, serum calcium, GWAS, Mendelian randomization, weighted median

## Abstract

It has been a long time that the relationship between serum calcium levels and Alzheimer’s disease (AD) remains unclear. Until recently, observational studies have evaluated the association between serum calcium levels and the risk of AD, however, reported inconsistent findings. Meanwhile, a Mendelian randomization (MR) study had been conducted to test the causal association between serum calcium levels and AD risk, however, only selected 6 serum calcium SNPs as the instrumental variables. Hence, these findings should be further verified using additional more genetic variants and large-scale genome-wide association study (GWAS) dataset to increase the statistical power. Here, we conduct an updated MR analysis of the causal association between serum calcium levels and the risk of AD using a two-stage design. In discovery stage, we conducted a MR analysis using 14 SNPs from serum calcium GWAS dataset (*N* = 61,079), and AD GWAS dataset (*N* = 63,926, 21,982 cases, 41,944 cognitively normal controls). All four MR methods including IVW, weighted median, MR-Egger, and MR-PRESSO showed a reduced trend of AD risk with the increased serum calcium levels. In the replication stage, we performed a MR analysis using 166 SNPs from serum calcium GWAS dataset (*N* = 305,349), and AD GWAS dataset (*N* = 63,926, 21,982 cases, 41,944 cognitively normal controls). Only the weighted median indicated that genetically increased serum calcium level was associated with the reduced risk of AD. Hence, additional studies are required to investigate these findings.

## Introduction

It has been a long time that the relationship between serum calcium levels and Alzheimer’s disease (AD) remains unclear (3–4), as few studies had investigated the association of serum calcium levels with AD (Deary et al., 1987; Landfield et al., 1991; Conley et al., 2009). Until recently, observational studies have evaluated the association between serum calcium levels and the risk of AD (Sato et al., 2019; Ma et al., 2021). However, these observational studies have highlighted inconsistent findings about the association of serum calcium levels with the risk of AD. Some observational studies have found the protective role of high serum calcium levels in AD (Deary et al., 1987; Landfield et al., 1991; Conley et al., 2009; Sato et al., 2019). Landfield et al. (1991) and Conley et al. (2009) found that AD cases had lower serum calcium levels compared with normal age-matched controls. Meanwhile, Shore et al. found that the severely demented patients had lower serum calcium levels compared with mildly affected individuals (Deary et al., 1987). Sato et al. (2019) analyzed the neuroimaging data of 234 mild cognitive impairment (MCI) participants from the Japanese Alzheimer’s Disease Neuroimaging Initiative (J-ADNI) study cohort. They found that low serum calcium levels could increase the conversion of MCI to early AD (Sato et al., 2019).

However, other observational studies have identified the harmful role of high serum calcium levels in AD. In a longitudinal population-based study, Kern et al. (2016) reported that compared with women without calcium supplementation, women with calcium supplements had increased risk of dementia and stroke-related dementia. Ma et al. (2021) analyzed the neuroimaging data of 1,224 non-demented elders including 413 cognitively normal and 811 MCI from ADNI. Their results indicated that serum calcium levels increased with the disease severity (Ma et al., 2021). High serum calcium could increase the cognitive decline and the conversion from non-demented status (cognitively normal and MCI) to AD (Ma et al., 2021).

In order to test the causal association between serum calcium levels and AD risk, He et al. (2020) conducted a Mendelian randomization (MR) study using genome-wide association study (GWAS) datasets from serum calcium and AD. He et al. (2020) found that genetically increased serum calcium levels could significantly reduce the risk of AD. This MR analysis still has two limitations. First, He et al. (2020) only selected 8 serum calcium related genetic variants as the potential instrumental variables. They further excluded two genetic variants using the pleiotropy analysis, and the remaining six genetic variants could only explain 0.81% of the serum calcium variance (He et al., 2020). Second, He et al. (2020) used four MR analysis methods including inverse-variance weighted (IVW), Weighted median, MR-Egger, and MR-PRESSO. However, the main analysis method IVW only indicated suggestive association (*P* = 0.031). Hence, these findings should be further verified using additional more genetic variants and large-scale GWAS dataset to increase the statistical power.

Until recently, large-scale GWAS of serum calcium levels (*N* = 305,349) and AD (*N* = 63,926, 21,982 cases, 41,944 cognitively normal controls) have been reported (Kunkle et al., 2019; Young et al., 2021). There GWAS included larger sample size than previous GWAS of serum calcium levels (*N* = 61,079) (O’seaghdha et al., 2013) and AD (*N* = 54,162, 17,008 AD cases and 37,154 controls) (Lambert et al., 2013), as used by He and colleagues, respectively (He et al., 2020). Importantly, these datasets are publicly available. Hence, we conduct an updated MR analysis of the causal association between serum calcium levels and the risk of AD using serum calcium GWAS datasets (O’seaghdha et al., 2013; Young et al., 2021), and AD GWAS dataset (Kunkle et al., 2019).

## Materials and Methods

### Study Design Overview

**FIGURE 1:**
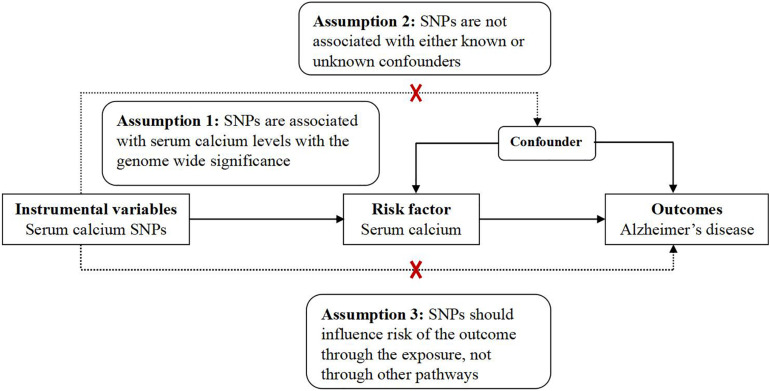
The framework of MR. MR analysis has three assumptions. Assumption 1: SNPs are associated with serum calcium levels with the genome wide significance; Assumption 2: SNPs are not associated with either known or unknown confounders; Assumption 3: SNPs should influence risk of the outcome through the exposure, not through other pathways.

This MR analysis is a two-sample MR study. Hence, we used the GWAS datasets from the exposure (serum calcium) and the outcome (AD) to estimate the effect of exposure on outcome (He et al., 2020). MR analysis has three assumptions, which have been widely described (Liu et al., 2018; Anderson et al., 2020; He et al., 2020; Wang L. et al., 2020; Zhang et al., 2020; Ou et al., 2021; Sproviero et al., 2021). Ethical approvals were provided in the original articles (Lambert et al., 2013; Young et al., 2021). Here, our MR analysis only used the GWAS summary datasets from serum calcium and AD (Lambert et al., 2013; Young et al., 2021). Hence, the informed consent is not needed. Figure 1 provides the framework of MR.

### Genetic Instrument Selection (Discovery)

In discovery stage, 14 serum calcium single nucleotide polymorphisms (SNPs) were selected including 8 SNPs at the genome-wide significance threshold (*P* < 5.00E-08), and 6 SNPs with *P* < 1.00E-04 (O’seaghdha et al., 2013). The 14 serum calcium SNPs were identified by a GWAS using 61,079 individuals of European descent (O’seaghdha et al., 2013). These 14 serum calcium SNPs, especially the 8 SNPs at the genome-wide significance threshold, have been widely used as the potential instrumental variables to evaluate the association of serum calcium with other human complex diseases or phenotypes (Larsson et al., 2017, 2019; Xu et al., 2017; Meng et al., 2020; Wang Y. et al., 2020; Qu et al., 2021; Sun et al., 2021; Young et al., 2021; Yuan et al., 2021). Detailed information about these 14 SNPs is presented in Supplementary Table 1.

### Genetic Instrument Selection (Replication)

In replication stage, 208 independent SNPs associated serum calcium levels at the genome-wide significance threshold (*P* < 5.00E-08) were identified by a recent GWAS using 305,349 individuals from the UK Biobank (Young et al., 2021). Compared with 7 SNPs explaining 0.9% of the total variance of total serum calcium, these 208 SNPs explain 5.8% of the total variance of total serum calcium (Young et al., 2021). Detailed information about these 208 SNPs is presented in Supplementary Table 2.

### AD GWAS Selection

The discovery GWAS summary statistics of AD were obtained from the International Genomics of Alzheimer’s Project (IGAP) stage 1 including 21,982 AD and 41,944 cognitively normal controls of European descent (Kunkle et al., 2019). The IGAP stage 1 is based the meta-analysis of four AD GWAS datasets including Alzheimer Disease Genetics Consortium, Cohorts for Heart and Aging Research in Genomic Epidemiology Consortium (CHARGE), The European Alzheimer’s Disease Initiative (EADI), and Genetic and Environmental Risk in AD/Defining Genetic, Polygenic and Environmental Risk for Alzheimer’s Disease Consortium (GERAD/PERADES) (Kunkle et al., 2019). AD cases were autopsy-confirmed or clinically confirmed using the NINCDS-ADRDA criteria or DSM-IV guidelines (Kunkle et al., 2019). The IGAP AD GWAS summary statistics have been widely used in recent MR analysis (Liu et al., 2018; Anderson et al., 2020; He et al., 2020; Wang L. et al., 2020; Zhang et al., 2020; Ou et al., 2021; Sproviero et al., 2021).

### MR Method Selection

Four MR methods were selected to evaluate the causal association between serum calcium and the risk of AD including the main analysis method inverse-variance weighted meta-analysis (IVW) (Bowden et al., 2016), and other three additional analysis methods weighted median (Bowden et al., 2016), MR-Egger (Burgess and Thompson, 2017), and Mendelian randomization pleiotropy residual sum and outlier (MR-PRESSO) (Verbanck et al., 2018). Meanwhile, MR-Egger intercept test and MRPRESSO Global test were used to evaluate the evidence of pleiotropy (Bowden et al., 2016; Burgess and Thompson, 2017; Verbanck et al., 2018; Bowden and Holmes, 2019). The odds ratio (OR) and 95% confidence interval (CI) of AD corresponds to 1 standard deviation (SD) in serum calcium levels. The statistical significance threshold was *P* < 0.05. All analyses were performed using R Version 4.0.3 and R packages (“MendelianRandomization”) and (“MRPRESSO”) (Yavorska and Burgess, 2017; Verbanck et al., 2018).

## Results

### MR Analysis in the Discovery Stage

**FIGURE 2:**
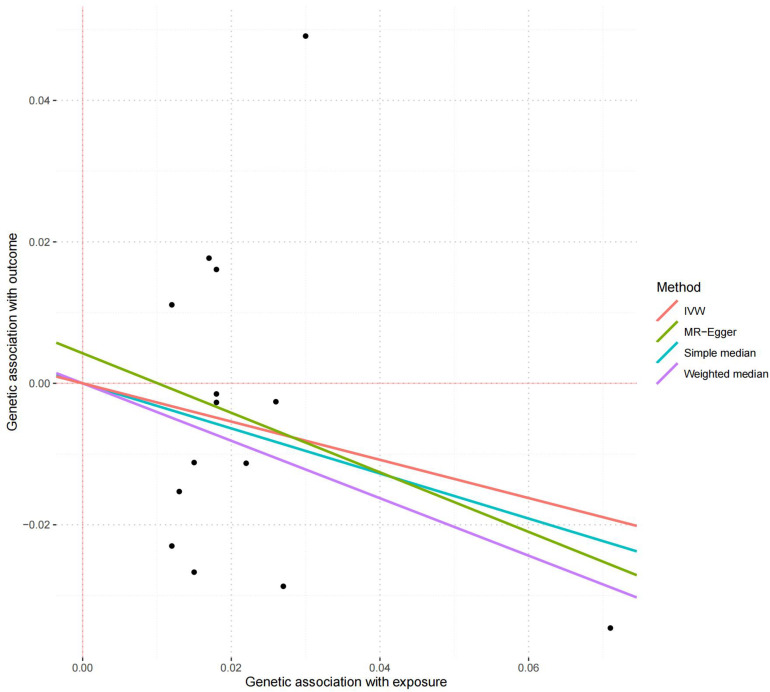
The scatter plot of the MR analysis in discovery stage using different methods. The scatter plot is based on the single causal estimates from 14 serum calcium SNPs using IVW, weighted median, simple median and MR-Egger, respectively. The scatter plot depicts the causal relationship between serum calcium level and the risk of AD. The *X*-axis stands for the effect estimate (beta coefficient) of serum calcium level utilizing a certain SNP; stands for the effect estimate (beta coefficient) of AD risk utilizing a certain IVW, Inverse variance weighting.

**FIGURE 3:**
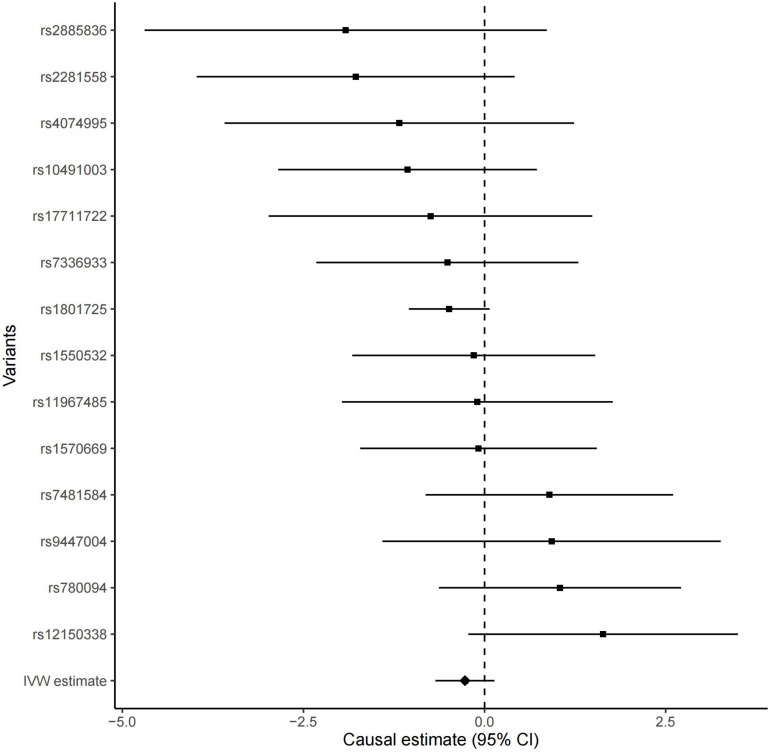
The forest plot of the single Mendelian randomization causal estimates for the association between genetically predicted serum calcium and the risk of AD from 14 serum calcium SNPs using IVW. The black point showed the causal effect estimate (beta coefficient) of serum calcium level on the risk of AD utilizing a certain SNP, and the black line indicated the 95% CI of the estimate. “IVW estimate” reports the effect using all SNPs estimated by the inverse-variance weighted method. CI, confidence interval.

**FIGURE 4:**
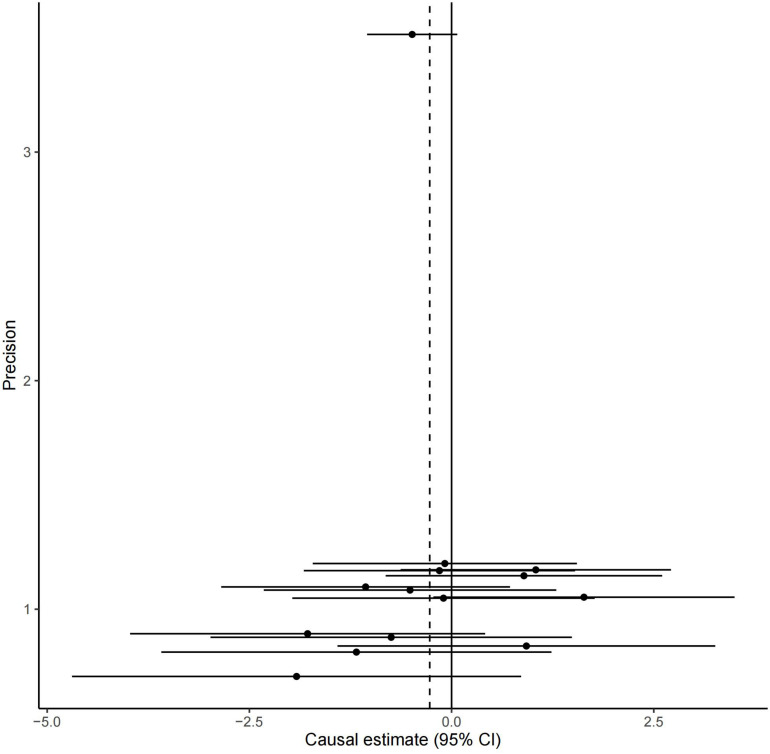
The funnel plot of the single causal estimates from 14 serum calcium SNPs using IVW. The funnel plot shows the potential bias of the selected 14 serum calcium SNPs. The *X*-axis stands for the causal effect estimate (beta coefficient) of serum calcium level on the risk of AD utilizing a certain SNP and the *Y*-axis is the reciprocal of standard error for each causal effect estimate. CI, confidence interval.

All these 14 serum calcium SNPs are available in the AD GWAS dataset. We then extracted their corresponding summary statistics for MR analysis, as provided in Table 1. The main and other additional MR methods indicated no significant association between serum calcium and the risk of AD including weighted median (OR = 0.67, 95% CI: 0.40–1.12, *P* = 1.22E-01), IVW (OR = 0.76, 95% CI: 0.51–1.15, *P* = 1.94E-01), MR-Egger (OR = 0.66, 95% CI: 0.30–1.42, *P* = 2.87E-01), and MR-PRESSO (OR = 0.76, 95% CI: 0.51–1.15, *P* = 2.17E-01), as provided in Table 2. However, all these four methods showed a reduced trend of AD risk with the increased serum calcium levels. Meanwhile, the MR-Egger intercept test (with intercept = 0.004, and *P* = 0.650) and MRPRESSO Global Test (*P* = 0.337) did not indicate evidence of pleiotropy. Figure 2 is the scatter plot of the single causal estimates from these 14 serum calcium SNPs using IVW, weighted median, simple median and MR-Egger. Figures 3, 4 are the forest plot, and funnel plot of the single causal estimates from these 14 serum calcium SNPs using IVW, respectively.

**TABLE 1:** Association of 14 serum calcium SNPs with AD risk.

**SNP**	**Serum calcium**						**AD**		
	**EA**	**NEA**	**EAF**	**Beta**	**SE**	***P* value**	**Beta**	**SE**	***P* value**
rs10491003	T	C	0.09	0.027	0.005	4.80E-09	−0.0287	0.0246	0.2442
rs11967485	G	A	0.9	0.026	0.005	9.40E-07	−0.0026	0.0248	0.9175
rs12150338	T	C	0.09	0.03	0.006	1.50E-06	0.0491	0.0285	0.08516
rs1550532	C	G	0.31	0.018	0.003	8.20E-11	−0.0027	0.0154	0.8593
rs1570669	G	A	0.34	0.018	0.003	9.10E-12	−0.0015	0.015	0.9188
rs17711722	T	C	0.47	0.015	0.003	8.20E-09	−0.0112	0.0171	0.5115
rs1801725	T	G	0.15	0.071	0.004	8.90E-86	−0.0346	0.0202	0.08741
rs2281558	T	G	0.25	0.015	0.003	5.10E-06	−0.0267	0.0168	0.1128
rs2885836	A	G	0.24	0.012	0.003	5.40E-05	−0.023	0.017	0.1759
rs4074995	A	G	0.28	0.013	0.003	4.60E-06	−0.0153	0.016	0.3385
rs7336933	G	A	0.85	0.022	0.004	9.10E-10	−0.0113	0.0203	0.5779
rs7481584	G	A	0.7	0.018	0.003	1.20E-10	0.0161	0.0157	0.3042
rs780094	T	C	0.42	0.017	0.003	1.30E-10	0.0177	0.0145	0.2216
rs9447004	A	G	0.48	0.012	0.003	3.30E-06	0.0111	0.0143	0.4387

*SNP, single-nucleotide polymorphism; EA, effect allele; NEA, non-effect allele; EAF, effect allele frequency; SE, standard error; Beta, regression coefficient based on the effect allele.*

**TABLE 2:** MR results in discovery and replication stages.

**Stage**	**Method**	**OR**	**95% CI**	***P* value**
Discovery	Weighted median	0.67	0.40–1.12	1.22E-01
Discovery	IVW	0.76	0.51–1.15	1.94E-01
Discovery	MR-Egger	0.66	0.30–1.42	2.87E-01
Discovery	MR-PRESSO Raw	0.76	0.51–1.15	2.17E-01
Discovery	MR-PRESSO Outlier-corrected	NA	NA	NA
Replication	Weighted median	0.15	0.02–0.90	3.80E-02
Replication	IVW	1.14	0.36–3.64	8.18E-01
Replication	MR-Egger	0.27	0.03–2.19	2.22E-01
Replication	MR-PRESSO Raw	1.15	0.36–3.64	8.19E-01
Replication	MR-PRESSO Outlier-corrected	0.85	0.30–2.39	7.62E-01

*OR, odds ratio; CI, confidence interval; IVW, Inverse-variance weighted meta-analysis.*

### MR Analysis in the Replication Stage

**FIGURE 5:**
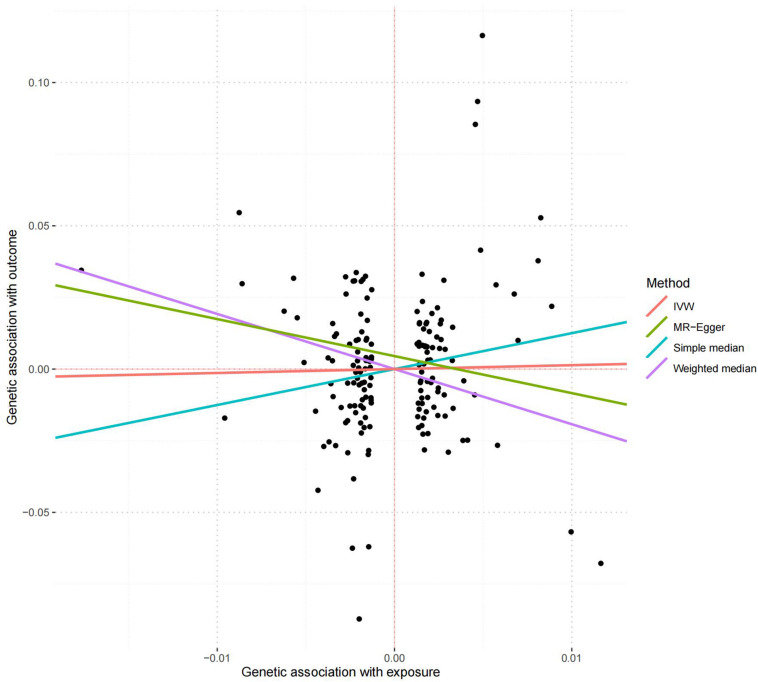
The scatter plot of the MR analysis in replication stage using different methods. The scatter plot is based on the single causal estimates from 166 serum calcium SNPs using IVW, weighted median, simple median and MR-Egger, respectively. The scatter plot depicts the causal relationship between serum calcium level and the risk of AD. The *X*-axis stands for the effect estimate (beta coefficient) of serum calcium level utilizing a certain SNP; stands for the effect estimate (beta coefficient) of AD risk utilizing a certain IVW, Inverse variance weighting.

166 of the 208 serum calcium SNPs are included in the AD GWAS dataset. We then extracted the summary statistics of these 166 SNPs for the MR analysis, as provided in Supplementary Table 3. Using the weighted median, we found that the genetically increased serum calcium level (per 1 SD increase) was associated with the reduced risk of AD (OR = 0.15, 95% CI: 0.02–0.90, *P* = 3.80E-02) (Table 2). However, the other MR methods did not reported any significant results including IVW (OR = 1.14, 95% CI: 0.36–3.64, *P* = 8.18E-01) and MR-Egger (OR = 0.27, 95% CI: 0.03–2.19, *P* = 2.22E-01). Meanwhile, MR-Egger intercept test did not indicate evidence of pleiotropy with intercept = 0.005, and *P* = 0.105. Using MRPRESSO, we found evidence of pleiotropy with Global Test *P* = 0.006. The MR-PRESSO Raw estimate is OR = 1.15, 95% CI: 0.36–3.64, *P* = 8.19E-01. The MR-PRESSO Outlier-corrected estimate is OR = 0.85, 95% CI: 0.30–2.39, *P* = 7.62E-01. Figure 5 is the scatter plot of the single causal estimates from these 166 serum calcium SNPs using IVW, weighted median, simple median and MR-Egger.

## Discussion

Calcium signaling is involved in many different intracellular and extracellular processes (Marambaud et al., 2009). It is known that AD is characterized by the extracellular accumulation of amyloid (Aβ) plaques and intracellular neurofibrillary tangles (NFTs) in the brain (Tong et al., 2018). Evidence shows that the calcium dysregulation occurs prior the key AD pathologies including plaques, tangles, and synaptic deficits (Tong et al., 2018). The disrupted calcium could further induce synaptic deficits, and promote the accumulation of Aβ plaques and NFTs (Tong et al., 2018). Hence, deregulated calcium homeostasis may play an important role in the pathogenesis of AD (Marambaud et al., 2009).

Until now, observational studies by analyzing the neuroimaging data have evaluated the association between serum calcium levels and the risk of AD, however, reported inconsistent findings (Sato et al., 2019; Ma et al., 2021). Sato et al. (2019) concluded that low serum calcium levels increased the conversion of MCI to early AD. Ma et al. (2021) found that high serum calcium increased the cognitive decline and the conversion from non-demented status (cognitively normal and MCI) to AD. Two reasons have caused these inconsistent findings. First, Sato et al. (2019) selected a total of 234 MCI individuals, and Ma et al. (2021) selected 413 cognitively normal and 811 MCI. Hence, the sample size may have affected the conclusions from both studies. Second, the samples used in both studies are of different descents including one from Japanese and the other European. Hence, different descents may have also affected the conclusions from both studies (Sato et al., 2019; Ma et al., 2021). Meanwhile, a longitudinal population-based study had tested the association between calcium supplementation and dementia in 700 dementia-free women aged 70–92 years (Kern et al., 2016). The results indicated that women with calcium supplements had higher risk of developing dementia than women without calcium supplementation (Kern et al., 2016). A cross sectional study in 337 subjects in India indicated that increased calcium level could increase the cognitive score (Basheer et al., 2016).

Here, we conduct an updated MR analysis of the causal association between serum calcium levels and the risk of AD using a two-stage design. In discovery stage, we conducted a MR analysis using 14 SNPs from serum calcium GWAS dataset (*N* = 61,079) (O’seaghdha et al., 2013), and AD GWAS dataset (*N* = 63,926, 21,982 cases, 41,944 cognitively normal controls) (Kunkle et al., 2019). All four MR methods including IVW, weighted median, MR-Egger, and MR-PRESSO showed a reduced trend of AD risk with the increased serum calcium levels. In the replication stage, we performed a MR analysis using 166 SNPs from serum calcium GWAS dataset (*N* = 305,349) (Young et al., 2021), and AD GWAS dataset (*N* = 63,926, 21,982 cases, 41,944 cognitively normal controls) (Kunkle et al., 2019). Only the weighted median indicated that genetically increased serum calcium level was associated with the reduced risk of AD, which indicates that 50% of the weight comes from the valid instrumental variables (Bowden et al., 2016; Bowden and Holmes, 2019). Our findings may have clinical application that high serum calcium level by diet or calcium supplementation may contribute to reduce the risk of AD. However, IVW, MR-Egger, and MR-PRESSO indicated no causal association between serum calcium level and the risk of AD. Hence, additional studies including MR studies and especially randomized controlled trials are required to investigate these findings.

Compared with the original MR study from He and colleagues, our MR analysis may have several strengths. First, we selected a large-scale AD GWAS dataset (*N* = 63,926, 21,982 cases, 41,944 cognitively normal controls) (Kunkle et al., 2019), which included more additional samples compared with the original study (*N* = 54,162, 21,982 cases, 41,944 cognitively normal controls), as used by He and colleagues (Lambert et al., 2013). Second, we selected 14 serum calcium SNPs in the discovery stage and 166 serum calcium SNPs in the replication stage. He and colleagues only selected six SNPs as the effective instrumental variables, and only observed suggestive association (*P* = 0.031) (He et al., 2020). Our undated MR analysis significantly increased the number of instrumental variables, which may contribute to the increases statistical power in MR analysis (He et al., 2020). Meanwhile, this two-sage method may contribute to test the replication and robustness of MR estimate. Third, the individuals from both the serum calcium and AD GWAS are of European descent. Hence, our MR analysis may have reduced the population stratification bias. Fourth, multiple MR and pleiotropy analysis methods including IVW, weighted median, MR-Egger, and MR-PRESSO were selected to reduce the pleiotropy.

Meanwhile, our MR study may also have some limitations. First, we selected 14 SNPs in the discovery stage, and 208 SNPs in the replication stage, as the potential instrumental variables. However, they are not completely in linkage disequilibrium. Hence, the linkage disequilibrium may have influenced the MR findings. Second, the 208 serum calcium SNPs are identified using is based UK Biobank samples (Young et al., 2021), and the AD GWAS dataset is based on the 21,982 AD and 41,944 cognitively normal controls of European descent (Kunkle et al., 2019). Hence, we could not ensure that the serum calcium GWAS dataset and AD GWAS dataset are completely independent with each other. Hence, the cryptic relatedness may have influenced the MR findings. Third, our MR findings are based on the individuals of European descent. Considering the genetic heterogeneity across the different descents, the MR findings between serum calcium levels and the risk of AD may be different. Hence, our findings are required to be tested in other populations. Fourth, we have evaluated the pleiotropy using both the MR-Egger intercept test and MRPRESSO test. However, we could not completely exclude all the pleiotropy. Hence, there may be other confounding factors, which may have influenced our MR findings. Hence, future studies are required to verify our findings.

## Conclusion

Collectively, our updated MR analysis highlighted a reduced trend of AD risk with the increased serum calcium levels in the discovery stage, and reduced risk of AD in the replication stage. Meanwhile, additional studies are required to investigate our findings.

## Data Availability Statement

The original contributions presented in the study are included in the article/Supplementary Material, further inquiries can be directed to the corresponding authors.

## Author Contributions

ZL and HL: conception and design. YS and RL: development of methodology and acquisition of data. YS, RL, YG, QL, HZ, and SY: analysis and interpretation of data, writing, review, and revision of the manuscript. All authors contributed to the article and approved the submitted version.

## Conflict of Interest

The authors declare that the research was conducted in the absence of any commercial or financial relationships that could be construed as a potential conflict of interest.

## Publisher’s Note

All claims expressed in this article are solely those of the authors and do not necessarily represent those of their affiliated organizations, or those of the publisher, the editors and the reviewers. Any product that may be evaluated in this article, or claim that may be made by its manufacturer, is not guaranteed or endorsed by the publisher.
