# Supplementary material for: An Updated Mendelian Randomization Analysis of the Association Between Serum Calcium Levels and the Risk of Alzheimer’s Disease
Source: Front Genet. 2021 Sep 8;12:731391. doi: 10.3389/fgene.2021.731391 (PMC8457382; doi:10.3389/fgene.2021.731391)
Supplement: Supplementary file 2 [file Table_1.DOCX]

**The programming codes for MR analysis using “MendelianRandomization”**

library(grid)

library(MendelianRandomization)

data <- read.table('F://data.txt')

mr <- mr_input(bx= data[,5], bxse = data[,6], by = data[,7], byse = data[,8], snps = data[,1])

mr_allmethods(mr)

**The programming codes for MR analysis using “MR-PRESSO”**

library(MRPRESSO)

SummaryStats <- read.table('F://data.txt', header=T)

mr_presso(BetaOutcome = "Y_effect", BetaExposure = "E1_effect", SdOutcome = "Y_se", SdExposure = "E1_se", OUTLIERtest = TRUE, DISTORTIONtest = TRUE, data = SummaryStats, NbDistribution = 2000, SignifThreshold = 0.05)
